# Thermogenic Targets for Obesity Management in the Era of Incretin-Based Therapies

**DOI:** 10.3390/ph18101519

**Published:** 2025-10-10

**Authors:** Sahar Soliman, Rebecca Andrews-Dickert, Petra Rocic, Mihail Mitov

**Affiliations:** Department of Physiology and Pharmacology, College of Osteopathic Medicine, Sam Houston State University, 925 City Central Avenue, Conroe, TX 77304, USA; sahar.soliman@shsu.edu (S.S.); rebecca.andrewsdickert@shsu.edu (R.A.-D.); pxr037@shsu.edu (P.R.)

**Keywords:** obesity pharmacotherapy, β3 receptors, β3 agonists, incretin-based therapies, thermogenic targets

## Abstract

The global rise in obesity continues to outpace advances in pharmacologic treatment. While incretin-based therapies have demonstrated significant efficacy in promoting weight loss, their widespread use remains limited by gastrointestinal side effects, long-term tolerability concerns, and access issues. Additionally, sustaining weight loss over time poses an ongoing clinical challenge. These limitations highlight the need for alternative or complementary pharmacologic strategies. One such approach involves stimulating thermogenesis, particularly through the activation of brown and beige adipose tissue. This narrative review focuses on β3 adrenergic receptors as key mediators of browning and thermogenic energy expenditure. We review preclinical and clinical data, address pharmacokinetic and delivery challenges, and assess the translational potential of targeting thermogenesis in the management of obesity. Future directions are proposed to guide the development of safe and effective therapies that utilize this underexplored pharmacologic pathway.

## 1. Introduction: Unmet Needs in Obesity Pharmacotherapy

Obesity has emerged as one of the most urgent public health challenges of the 21st century, with its prevalence rising rapidly across both developed and developing nations. In North America, Center for Disease Control and Prevention data from 2023 to 2024 reveal that in 23 U.S. states, more than one in three adults (35%) are classified as obese. This indicates an alarming shift given that, prior to 2013, no state had an adult obesity rate at or above this threshold. Currently, at least one in five adults (20%) in every U.S. state is living with obesity, with notable disparities by age, race, and socioeconomic status [1]. Globally, the World Health Organization reports that adult obesity rates have more than doubled since 1990, now affecting approximately 16% of the global adult population [2]. Childhood obesity has also surged, increasing fourfold among individuals aged 5–19 years during the same period [3]. Obesity is a major risk factor for numerous noncommunicable diseases, including cardiovascular disease [4], type 2 diabetes [5], and several types of cancer [6], contributing to millions of preventable deaths each year [7].

These trends highlight the urgent need for comprehensive, evidence-based strategies to address the complex, multifactorial causes of obesity and reduce its growing burden on healthcare systems worldwide.

Incretin-based therapies have revolutionized the management of type 2 diabetes and obesity by harnessing the physiological actions of gut-derived hormones such as glucagon-like peptide-1 (GLP-1) and glucose-dependent insulinotropic polypeptide (GIP). These hormones enhance glucose-dependent insulin secretion and contribute to appetite regulation and weight loss, making them attractive targets for pharmacological intervention [8,9]. The development of potent GLP-1 receptor agonists (GLP–1 RAs) such as semaglutide and dual agonists for both GLP-1 and GIP, like tirzepatide, has led to unprecedented clinical outcomes, including significant weight reductions and significant improvements in cardiometabolic health [8]. Recent trials, such as those evaluating mazdutide, further indicate the efficacy of these agents in diverse populations, including individuals with obesity and related comorbidities [10]. However, drawbacks of GLP-1 agonist pharmacotherapy include tolerability, especially at higher doses, and association with rare but serious adverse effects [11], which necessitates exploring new avenues as alternative agents, or in combination with lower doses of existing FDA-approved agents.

Various studies suggest that incretin-based therapies may also modulate energy expenditure pathways and potentially enhance thermogenesis in adipose tissue [12]. In studies using Swiss mice and Sprague Dawley rats, central activation of the GLP-1 receptor (GLP-1R) by liraglutide results in body weight loss that occurs independently of reduced food intake. This effect is driven by the activation of thermogenic programs in brown adipose tissue (BAT), with targeted injection of liraglutide into the ventromedial hypothalamus sufficient to induce weight loss and stimulate BAT thermogenesis through 5′adenosine monophosphate-activated protein kinase (AMPK)-dependent signaling [13].

These findings suggest a potential role for combining incretin-based treatments with thermogenic approaches that influence mitochondrial uncoupling—a process in which energy is released as heat rather than stored as adenosine triphosphate (ATP). Agents that activate brown adipose tissue (BAT) or induce browning of white adipose tissue (WAT) through uncoupling proteins such as thermogenin, also known as uncoupling protein 1 (UCP1) are under investigation as possible ways to increase energy expenditure [14]. Integrating appetite regulation through incretin signaling with metabolic enhancement via thermogenesis may offer complementary benefits in the management of obesity and metabolic syndrome. Nevertheless, even with the success of GLP-1 receptor agonists and dual agonists such as tirzepatide, important gaps remain in obesity pharmacotherapy—particularly the limited engagement of thermogenic mechanisms. Below, we summarize several of these unmet needs.

### 1.1. Plateau in Weight Loss Despite Continued Incretin Therapy

**Figure 1:**
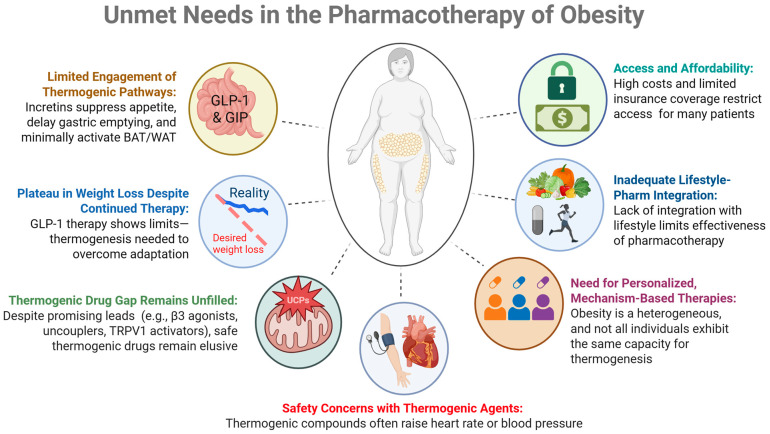
Summary of the current limitations and barriers to the existing incretin therapies.

GLP-1 RAs, such as liraglutide and semaglutide, effectively promote weight loss by suppressing appetite and delaying gastric emptying. However, many patients reach a weight loss plateau over time, suggesting that appetite regulation alone may be insufficient for achieving and maintaining a desired weight loss (Figure 1). For example, in a 2-year study of liraglutide, patients initially experienced significant weight loss, especially at the 3.0 mg dose. However, after the first year, weight loss tended to plateau despite continued therapy. The results of this suggest that while GLP-1 RAs are effective in the short term, their long-term efficacy may diminish, possibly due to physiological adaptations [15]. A key study published in the New England Journal of Medicine demonstrated that while liraglutide alone was more effective than placebo or exercise alone in maintaining weight loss after an initial low-calorie diet, the combination of liraglutide and structured exercise produced the most substantial and sustained weight reduction. Participants in the combination group not only lost more weight but also experienced greater improvements in body composition, insulin sensitivity, and cardiorespiratory fitness. These findings suggest that GLP-1 RAs like liraglutide may reach a plateau in their effectiveness when used alone, targeting mechanisms that increase energy expenditure may complement GLP-1 RAs and result in additional weight loss [16]. Pharmacological agents that promote thermogenesis, thereby increasing energy expenditure while preserving lean mass, may further complement GLP-1 RAs. As a result, research is now shifting toward identifying potential combination therapies that boost thermogenesis to overcome the limitations of expenditure (such as exercise-induced thermogenesis) and appear to reduce mortality.

### 1.2. Limited Engagement of Thermogenic Pathways During Incretin Monotherapies

The effects of GLP-1 and GIP on obesity, thermogenesis, and adipocyte activation are complex and modulated by several physiological and experimental variables [17]. These include the route of administration and site of action [18,19], dosage [20], species differences (rodents vs. humans) [21], and metabolic context, such as obesity or type 2 diabetes [22]. GLP-1 RAs have demonstrated the ability to stimulate thermogenesis in BAT and promote the browning of WAT, particularly in rodent models [13,23,24]. These effects are primarily mediated through central GLP-1R activation in the hypothalamus, which enhances sympathetic nervous system output to adipose tissues [25,26]. Additionally, preclinical studies show that GLP-1/GLP-1R signaling promotes preadipocyte proliferation and differentiation while lowering glycemia and attenuating weight gain [27,28], suggesting metabolically favorable adipose remodeling rather than net adipose accumulation. Clinically, GLP-1RAs reduce both visceral (VAT) and subcutaneous adipose (SAT) depots to a similar absolute extent (meta-analysis mean changes ~−21 cm^2^ VAT and ~−23 cm^2^ SAT), with depot losses tracking overall weight reduction rather than showing strong BAT/WAT selectivity [29].

The glucose-dependent insulinotropic polypeptide (GIP) is a 42-amino-acid incretin released from proximal duodenal K cells after fat or glucose ingestion [30,31]. In mice, GIPR mRNA is detectable across multiple white depots: mesenteric, inguinal, epididymal, and retroperitoneal, and in interscapular brown fat. In BAT, GIP signaling modulates thermogenic gene programs, fuel utilization, and oxygen consumption; however, BAT-specific GIPR deletion does not reproduce the reduced weight gain observed with systemic GIP disruption, suggesting extra-BAT sites of action [32]. Single-cell/-nucleus and integrative analyses further indicate that in mouse WAT most GIPR signal resides in stromal–vascular and immune compartments rather than mature adipocytes, with enrichment in pericytes and mesothelial cells, consistent with effects of GIP on lipolysis/lipid storage, glucose handling, insulin sensitivity, adipokines, blood flow, and inflammatory tone [33]. Functionally, adipocyte-targeted GIPR induction in mice increases lipid oxidation and thermogenesis via sarcoplasmic/endoplasmic reticulum Ca^2+^-ATPase (SERCA)-mediated futile Ca^2+^ cycling, resulting in ~35% weight loss with persistence after transgene switch-off, thereby conferring a durable metabolic memory [34]. In humans, GIPR is low/undetectable in preadipocytes but is induced with adipocyte differentiation, where it couples to cAMP signaling and lipoprotein lipase activity; tissue-level GIPR mRNA has been reported in subcutaneous and visceral fat [35].

The development of dual GLP-1/GIP receptor agonists, such as tirzepatide, represents a promising therapeutic strategy. These agents leverage the synergistic actions of both incretins, resulting in greater weight loss, improved glycemic control, and enhanced adipose tissue function compared to GLP-1 RAs alone. In Lilly’s SURMOUNT-1 trial (international, phase 3, double-blind, randomized, placebo-controlled trial of tirzepatide in persons with obesity), tirzepatide achieved up to a ~21% reduction in body weight over 72 weeks in people with obesity, significantly outperforming placebo [36].

### 1.3. Therapeutic Void (Lack of FDA-Approved Agents Specifically Targeting Thermogenesis)

In obesity pharmacotherapy, preclinical thermogenic drug candidates targeting multiple mechanisms (listed below in Section 1.3.1, Section 1.3.2, Section 1.3.3, Section 1.3.4 and Section 1.3.5) have demonstrated potential for activating BAT [37,38].

Initially, BAT in humans was thought to be primarily active in infants, with neonates having an interscapular BAT depot that regressed with age [39,40,41]. However, autopsies and studies using ^18^F-fluorodeoxyglucose positron emission tomography/computerized tomography demonstrated that adults retain active BAT in well-defined regions [40,42,43,44,45,46,47,48]. Constitutive “brown” adipocytes are located in the cervical, supraclavicular, and paravertebral areas, whereas recruitable “beige” (brite) adipocytes can be found in supraclavicular, abdominal, and other smaller depots [39,44,49,50,51]. More detailed anatomical mapping shows visceral BAT around the aorta, coronary vessels, mediastinum, abdominal organs, and subcutaneous BAT between the neck muscles, supraclavicular fossa, axilla, anterior abdominal wall, and inguinal fossa [52]. Although the absolute amount is small, estimates suggest that as little as 50 g of maximally stimulated BAT could account for up to 20% of daily energy expenditure in adults [53]. Thus, drug candidates that target thermogenesis have the potential to be beneficial in weight management. However, their clinical translation has been hindered by concerns over efficacy, safety [54,55].

#### 1.3.1. β3-Adrenergic Receptor Agonists

These agents stimulate β3-adrenergic receptors (β3-ARs), which are highly expressed in BAT and play a central role in lipolysis and thermogenesis [56]. In rodent models, β3-AR agonists effectively increase energy expenditure and promote WAT browning [57]. However, human trials have been less successful, in part due to lower β3-receptor density in adult human BAT [58] and the emergence of off-target cardiovascular effects, such as increased heart rate and blood pressure [59] (see Section 1.4 for additional details).

#### 1.3.2. Mitochondrial Uncouplers

Compounds such as 2,4-dinitrophenol (DNP) increase energy expenditure by uncoupling oxidative phosphorylation, thereby dissipating the proton gradient in mitochondria and generating heat instead of ATP [60]. While potent, DNP and similar compounds have historically been associated with severe toxicity, hyperthermia, and narrow therapeutic windows that preclude safe use [61,62]. Efforts to develop newer, liver-targeted or controlled-release uncouplers are ongoing [63], with some early-stage candidates (such as BAM15) showing improved safety profiles [64], but none have reached late-phase clinical testing [65].

#### 1.3.3. TRPV1 Agonists

Transient receptor potential vanilloid 1 (TRPV1) agonists (such as capsaicin) activate sensory neurons, stimulate sympathetic outflow, and enhance BAT activity [66,67]. Preclinical studies suggest potential benefits in both BAT thermogenesis and WAT browning [68,69]. However, in humans, gastrointestinal discomfort, poor tolerability, and variable oral bioavailability have limited their utility as long-term therapeutic agents [70].

#### 1.3.4. Pharmacological Activation of Thyroid Hormone Receptors

Thyroid hormone receptor (THR) agonists can induce thermogenesis in adipose tissue by promoting mitochondrial biogenesis and increasing uncoupled respiration [71]. A synthetic THR agonist has been shown to stimulate browning of subcutaneous WAT and enhance energy expenditure in rodent models, offering an alternative to β3-adrenergic–mediated pathways [72]. Unlike β3-RAs, which rely on adrenergic receptor density, THR activation initiates thermogenic programs independently [73]. Centrally, thyroid hormone (TH) enhances thermogenesis through the sympathetic nervous system (SNS) [74], while peripherally it acts directly on BAT. Similarly to adaptive thermogenesis, TH promotes glucose uptake and stimulates lipolysis to provide substrates for β-oxidation. In addition, TH directly upregulates UCP1 expression in BAT, further supporting its thermogenic role [75]. However, clinical translation will require careful evaluation of safety concerns due to the risk of thyrotoxic effects.

#### 1.3.5. Centrally Acting Sympathomimetics

Centrally acting sympathomimetics, including amphetamine derivatives (e.g., phentermine), have been used in practice for weight loss for decades. Amphetamines act primarily through hypothalamic catecholaminergic appetite suppression, and their effects on energy expenditure and thermogenesis are variable across human and animal studies [76,77,78,79,80,81]. Importantly, direct evidence of phentermine activating human BAT or inducing WAT browning is lacking. Preclinical findings are mixed, with ~35% higher energy expenditure in mice, suggesting a thermogenic component [82], but reduced UCP1 expression in rat BAT during sustained administration, arguing against a direct BAT-activating mechanism [83].

Despite decades of research, no FDA-approved drugs currently exist that directly activate BAT or induce WAT browning in a safe, effective, and sustained manner [84]. This gap highlights the need for therapies that harness adipose thermogenesis, via BAT activation and/or WAT browning, that could be deployed as stand-alone approaches or, pending supportive evidence, in combination with established intake-focused treatments. Additionally, and beyond canonical UCP1-driven pathways, UCP1-independent mechanisms, including ATP-linked creatine, lipid, and Ca^2+^ substrate futile cycles, are gaining traction as promising future targets to raise energy expenditure [85,86,87]. Although pharmacologic tools are still in the early stages, these mechanisms represent additional avenues under investigation for obesity treatment.

### 1.4. Safety Concerns About Thermogenic Agents

Many thermogenic compounds have been limited by cardiovascular or systemic side effects, such as increased heart rate or blood pressure (see Table 1 for details). This highlights the need for selective, tissue-specific agents that can safely target thermogenic pathways to activate BAT or induce WAT browning without triggering undesirable off-target effects.

### 1.5. Need for Personalized and Mechanism-Based Therapies

GLP-1 agonists are now widely prescribed for obesity management; however, their utility may be constrained by side effects, including common gastrointestinal side effects, such as nausea, vomiting, diarrhea, and constipation [103]. More concerning adverse effects associated with GLP-1 agonists include pancreatitis, biliary disease, and bowel obstruction [11]. Use of GLP-1 agonists can also lead to muscle mass loss due to decreased calorie and protein intake [104]. Additionally, a family or personal history of medullary thyroid cancer is a contraindication to GLP-1 agonists due to an increased risk of thyroid C-cell tumors found in rodent studies [105]. For populations unable to tolerate GLP-1 receptor agonists or with contraindications to their use, a personalized approach and alternative strategies tailored to individual needs may offer greater benefit.

Additionally, the pharmacological management of obesity has focused mainly on adult populations, leaving a significant gap in evidence-based options for children and adolescents. This is particularly concerning given the alarming rise in pediatric obesity, which now affects over 20% of adolescents in many developed countries and is increasingly prevalent worldwide [106].

Pharmacotherapy for pediatric obesity remains limited [107], with few approved agents and insufficient long-term safety data, highlighting a critical need for age-specific research and therapeutic development.

In the United States, approximately one-third of children and adolescents have excess body weight (overweight or obesity), and globally the prevalence has risen across most resource-abundant settings over the last five decades [108,109]. Projections estimate 206 million youths living with obesity by 2025 and 254 million by 2030, concentrated not only in high-income countries but also in large middle-income nations [106,110]. Even where overall prevalence has plateaued, severe pediatric obesity is increasing, often accounting for a quarter of cases in European surveillance, and is associated with socioeconomic disadvantage in high-income settings [111,112,113,114,115,116]. The COVID-19 pandemic further accelerated unhealthy weight gain trajectories in multiple cohorts. Importantly, childhood adiposity tracks into adulthood, and tracking is related to age at onset, severity of obesity, and childhood BMI trajectory, as well as parental obesity [117,118,119,120,121,122,123,124]. These observations support expanding pediatric anti-obesity pharmacotherapy investigations, alongside lifestyle interventions, as a strategy to mitigate adult obesity.

Since the American Academy of Pediatrics (AAP) first opened the door to pediatric pharmacotherapy within tertiary care in its 2007 guidelines [125], the treatment landscape has evolved in stages: earlier agents included sibutramine (withdrawn in 2010) and orlistat (limited by tolerability). More recently, the approval of phentermine–topiramate ER was expanded to adolescents ≥ 12 years in 2022 [126,127]. In January 2023, the AAP recommended offering pharmacotherapy as an adjunct to lifestyle for youth ≥ 12 years, with selective use in 8–11 years [128]. Despite these steps, available pediatric medications and pharmacotherapeutic options remain limited. Lifestyle modifications remain foundational, although sustaining balanced nutrition, activity, and sleep can be difficult in obesogenic environments [129].

The most robust pediatric data now derive from GLP-1 receptor agonists (GLP-1RAs). Two randomized, placebo-controlled trials in adolescents demonstrated clear efficacy: SCALE Teens (liraglutide, 56 weeks) showed modest BMI reduction and higher response rates than placebo [130], while STEP TEENS (semaglutide, 68 weeks) achieved a –16% mean BMI change, with most participants reaching clinically meaningful weight loss [131]. In younger children (SCALE Kids, ages 6–<12), liraglutide plus lifestyle reduced BMI compared with continued weight gain on placebo [132]. Across trials, gastrointestinal events were common during dose escalation (≈63–65% with GLP-1RAs vs. 37–42% with placebo), with few discontinuations; growth, bone age, and pubertal development did not differ from placebo over ~1 year, but longer-term safety remains [133] undefined [126,130,131].

Despite these advances, three significant gaps remain. First, pediatric obesity continues to rise globally, with pandemic-era accelerations worsening the trend [106,133,134]. Second, only a few agents are approved for youths, leaving limited alternatives for those who cannot tolerate current therapies. Third, long-term safety and durability data are lacking [126].

Because children generally have a greater BAT volume and activity than adults, and BAT declines with age [39,40,41], pediatric care is uniquely positioned to leverage thermogenesis while BAT potential is at its highest. These considerations support further exploration of thermogenic agents that target BAT, either as new options or as complements to appetite-directed therapies in pediatric obesity.

Obesity is a heterogeneous disease [135]. Individual variability in both the capacity for thermogenesis [135,136] and intolerance to GLP-1–based therapies highlights the need for a personalized approach to obesity pharmacotherapy, emphasizing the importance of developing multiple therapeutic strategies that can be tailored to individual needs and extended to diverse populations, including children.

### 1.6. Inadequate Integration with Lifestyle Interventions

Pharmacotherapy is most effective when combined with lifestyle modification [137]. However, in real-world settings, this integration is often lacking. There is a need for structured, multidisciplinary approaches that combine medication with behavioral support, dietary counseling, and physical activity [138].

### 1.7. Access and Affordability

Access to effective obesity pharmacotherapy is significantly hindered by high costs and limited insurance coverage, disproportionately affecting underserved populations and exacerbating health disparities. As highlighted in some literature reports [139], such economic barriers restrict the reach of newer, more effective treatments like GLP-1 RAs, despite their clinical benefits. Addressing affordability and coverage is essential to ensure equitable obesity care across all demographics.

## 2. β3-Adrenergic Receptors and the Mechanism of Thermogenesis

**Figure 2:**
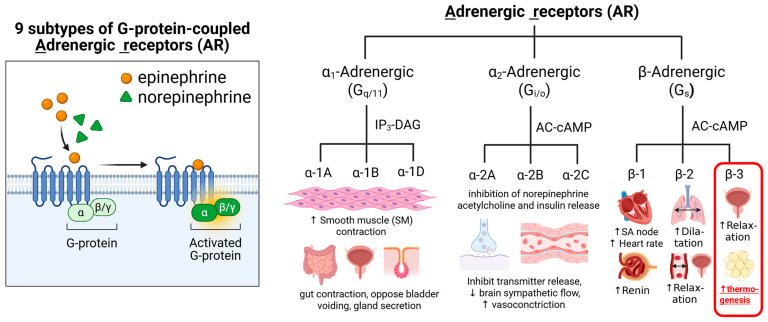
Summary of Adrenergic Receptors: Types, mechanisms of action, target tissues, and main known physiological effects.Gq is the most commonly studied subclass of the Gq/11 subfamily, Gi/o protein is Gi alpha subunit or Go alpha subunit, representing the alpha subunits of the Gi (inhibitory) and Go (other) families of heterotrimeric G proteins. IP3 (inositol trisphosphate) and DAG (diacylglycerol) are key second messenger molecules. Gs protein is Stimulatory Guanine Nucleotide Binding Protein. Gs is a type of G protein that stimulates the activation of cellular processes by coupling with certain receptors to increase cyclic AMP (cAMP) formation, which is a crucial second messenger in many signaling pathways.

Generally, all known adrenergic receptors are part of a family of G protein-coupled receptors (GPCR) that are activated by catecholamines (i.e., epinephrine and norepinephrine). The nine subtypes of adrenergic receptors (α1A, α1B, α1D, α2A, α2B, α2C, β1, β2, and β3) can be divided into two main classes of adrenergic receptors, the α-adrenergic and the β-adrenergic families [140,141]. Both types of adrenergic receptors were distinguished initially by their ligand binding characteristics (reactions with agonists and antagonists). All adrenergic receptors are involved in the autonomic nervous system, specifically in the sympathetic nervous system [142]. The α-adrenergic receptors primarily mediate vasoconstriction and smooth muscle contraction, while β-adrenergic receptors are involved in vasodilation, heart rate regulation, and other effects on smooth muscle (Figure 2).

The β3-ARs are one of the subtypes of adrenergic receptors primarily expressed in adipose tissue, including BAT [56,143]. They are involved in regulating lipolysis and thermogenesis and are also found in other tissues, such as the urinary bladder, gallbladder, and heart. β3-AR activation can increase energy expenditure, fat breakdown, and improve insulin sensitivity, making it a potential therapeutic target for conditions like obesity and type 2 diabetes. Due to their role in regulating lipolysis and thermogenesis, β3-ARs have been explored as potential therapeutic targets for obesity, type 2 diabetes, and overactive bladder syndrome. Several drugs that selectively target β3-adrenergic receptors (β3-ARs) have been identified, including agonists (which activate the receptor) and antagonists (which block the receptor). For example, mirabegron is a selective β3-AR agonist used to treat patients with an overactive bladder [144].

**Figure 3:**
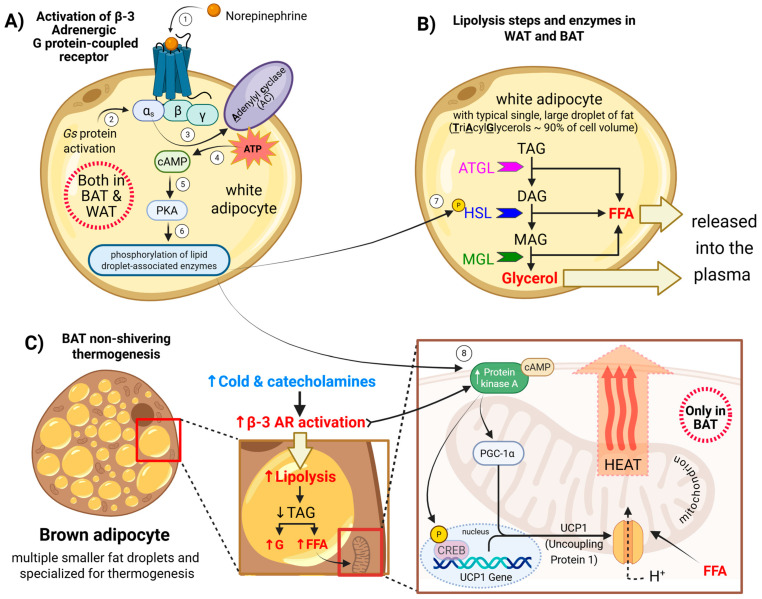
Mechanisms of β3-adrenergic receptor (β3-AR) activation leading to lipolysis and thermogenesis: (**A**) β3-ARs-Gs-cAMP-PKA pathway. (**B**) PKA-driven lipolysis releases FFA and glycerol. (**C**) FFAs fuel β-oxidation and activate UCP1; PKA–CREB upregulates thermogenic genes. Arrows and numbers in the figure indicate the sequence of events in β3-adrenergic receptor signaling. The color scheme distinguishes factors influencing β3-receptor activation: blue represents cold exposure and indicates lower temperature, while red denotes β3-receptor activation leading to increased heat production (enhanced thermogenesis) and/or elevated lipolysis, which provides substrates that support thermogenic processes.

The known mechanisms of β3-AR activation that lead to lipolysis and thermogenesis involve the following steps: Norepinephrine released from the sympathetic nervous system initiates β3-AR activation by coupling to the stimulatory G protein (Gs) [145]. These changes in Gs activate the enzyme adenylyl cyclase (AC), which converts ATP into the second messenger cyclic adenosine monophosphate (cAMP). Elevated cAMP levels activate protein kinase A (PKA), which in turn activates various target proteins to initiate the processes of lipolysis and thermogenesis (Figure 3A).

In white and brown adipocytes, PKA activation promotes lipolysis by a series of phosphorylation events that lead to the breakdown of stored triglycerides into fatty acids and glycerol. PKA phosphorylates perilipin, a protein on the surface of lipid droplets. This causes perilipin to change its shape, allowing enzymes to access the stored fat inside the droplet [146]. PKA also phosphorylates and activates hormone-sensitive lipase (HSL) and stimulates the activity of adipose triglyceride lipase (ATGL) through its cofactor, CGI-58 [147]. These lipases work together to hydrolyze the triglycerides, and as triglycerides are broken down, they release free fatty acids (FFAs) and glycerol. In brown and beige fat, these FFAs are used to fuel thermogenesis (Figure 3B).

The lipolysis triggers the enzymatic breakdown of triglyceride stores into glycerol and free FFAs [148]. The FFAs serve dual roles: as substrates for β-oxidation and as allosteric activators ofUCP1 in BAT mitochondria. Within mitochondria, UCP1 dissipates the proton gradient across the inner membrane by allowing protons to re-enter the matrix without driving ATP synthesis [149]. This process, known as non-shivering thermogenesis, converts the stored electrochemical energy into heat. In parallel, PKA phosphorylates and activates the transcription factor cAMP-responsive element-binding protein (CREB), upregulating thermogenic genes such as UCP1 and Peroxisome Proliferator-Activated Receptor Gamma Coactivator 1-Alpha (PGC-1α), a master regulator of mitochondrial biogenesis and thermogenic programming, which are essential for adaptive BAT responses to cold exposure. Thus, β3-AR signaling links catecholamine stimulation to enhanced energy expenditure through both substrate mobilization and mitochondrial uncoupling.

## 3. β3-Adrenergic Receptor Agonists: FDA-Approved Indications

The β3-adrenergic receptor (β3-AR) was not cloned until 1989, but it became apparent shortly thereafter that β3-AR agonists might hold therapeutic potential for obesity [55]. These receptors are increasingly recognized as compelling targets for obesity management due to their ability to stimulate thermogenesis [48] and promote adipose tissue remodeling [59]. β3-ARs are primarily expressed in adipose tissue, where activation of the Gs protein–coupled receptor pathway elevates intracellular cAMP, triggering protein kinase A (PKA)-dependent signaling and promoting lipolysis and thermogenic activity [56,150]. Experimental silencing of β3-AR has been shown to downregulate genes essential for thermogenesis, fatty acid metabolism, and mitochondrial biogenesis, resulting in reduced β3-agonist–induced cAMP production, lipolytic activation, and UCP1-mediated thermogenic capacity [56]. Although most abundant in adipose tissue, β3-AR expression has also been documented in the urinary bladder, gallbladder, and gastrointestinal tract [151], and clinical drug development has largely focused on these tissues, with FDA-approved β3 agonists currently indicated for overactive bladder rather than metabolic disorders [152,153,154]. Nonetheless, the selective distribution and downstream metabolic effects of β3-ARs continue to position them as attractive candidates for therapeutic intervention in obesity and related metabolic diseases. At present, no β3-AR agonist is FDA-approved or used in routine clinical practice for weight loss; evaluation of these agents for obesity remains investigational.

Mirabegron, a selective human β3-adrenergic receptor agonist, was the first β3 agonist approved for clinical use, receiving FDA approval in 2012 for the treatment of overactive bladder under the brand name Myrbetriq [155]. Administered orally, mirabegron may be used as monotherapy or in combination with antimuscarinic agents to relax the detrusor muscle during the storage phase of micturition, thereby increasing bladder capacity (according to the Mirabegron FDA label (https://www.accessdata.fda.gov/drugsatfda_docs/label/2018/202611s011lbl.pdf, accessed on 3 October 2025)). Beyond its urological indication, mirabegron has drawn attention for its metabolic potential; preclinical and translational studies have shown that it can stimulate lipolysis and thermogenesis via β3-AR activation in BAT. Such effects were abolished after silencing β3-AR expression [56]. These observations have revived interest in repurposing mirabegron for obesity treatment. Importantly, mirabegron is not approved for body-weight reduction, and its study in obesity to date has been limited to small, short-duration proof-of-concept investigations; it is not used in routine obesity care.

In a landmark human study, Cypess et al. demonstrated that a single 200 mg dose of mirabegron increased BAT metabolic activity as measured by ^18^F-FDG uptake, raised resting energy expenditure, and elevated plasma insulin and glucose levels, suggesting enhanced BAT activity and potential metabolic benefits in healthy individuals [48]. More recently, Dabrowska and Dudka reviewed both clinical and experimental evidence supporting mirabegron’s ability to promote adipose browning, improve glucose tolerance, and modulate lipid profiles, proposing that β3-AR agonists like mirabegron may represent a novel strategy for metabolic intervention [156]. Together, these findings provide a strong rationale for evaluating mirabegron’s utility beyond its current indications.

Despite growing interest in the metabolic applications of mirabegron, its clinical utility beyond urology remains constrained by dose-dependent cardiovascular effects [157,158]). At approved therapeutic doses (25–50 mg/day), mirabegron has been associated with modest elevations in systolic blood pressure and heart rate, along with reports of palpitations, tachycardia, and occasional exacerbation of preexisting hypertension [159,160]. These hemodynamic changes may emerge within hours of administration and have been observed during the initial days of treatment [158]. Although large-scale analyses have not shown a significant increase in major adverse cardiovascular events, such as stroke or myocardial infarction [161,162], these findings do not preclude concern for long-term use in obesity, which often co-exists with cardiovascular comorbidities and requires long-term pharmacologic therapy. At supratherapeutic doses (e.g., 200 mg/day), mirabegron has been associated with QTc interval prolongation, likely driven by off-target β1-adrenergic receptor stimulation at higher plasma concentrations [163,164,165]. Intermediate dosing regimens have shown some promise; a 100 mg dose was found to increase energy expenditure and supraclavicular skin temperature in a β3-selective manner, without concurrent elevations in heart rate or blood pressure [159]. These findings underscore the importance of defining an optimal therapeutic window for β3-adrenergic receptor agonists; one that preserves metabolic efficacy while limiting cardiovascular risk. Some reports proposed mitigation strategies such as the co-administration with β1-blockers and the exploration of chronic low-dose regimens aimed at sustaining brown and beige adipose tissue activation with improved tolerability [156].

Vibegron, marketed as Gemtesa, was approved by the FDA in December 2020 for the treatment of overactive bladder [166]. Like mirabegron, it is a selective β3-adrenergic receptor agonist; however, emerging evidence suggests that it may offer pharmacologic and safety advantages that make it a more attractive candidate for long-term metabolic applications. As with mirabegron, vibegron is not authorized for weight loss and has not been adopted for obesity treatment; investigations of metabolic effects remain confined to research settings. Nonetheless, given its near-exclusive β3 selectivity, negligible β1/β2 activity, preserved intrinsic activity at low receptor density, and a more favorable safety/tolerability profile, we propose that vibegron may yield superior metabolic/thermogenic outcomes compared with mirabegron, pending confirmation in adequately powered obesity trials.

In a comparative pharmacologic evaluation of four β3 agonists (vibegron, mirabegron, and the investigational agents solabegron and ritobegron), vibegron demonstrated the highest selectivity, showing over 7900-fold preference for β3- over β1- and β2-adrenergic receptors. It exhibited no measurable agonist activity at β1- or β2-receptors, even at receptor densities relevant to human cardiac tissue, supporting a favorable cardiovascular safety profile [167]. In addition to its superior selectivity, vibegron retained full intrinsic agonist activity even under conditions of low β3 receptor density. This characteristic may be particularly advantageous in adult humans [167], where BAT expresses β3-adrenergic receptors at much lower levels than in rodent models. Vibegron also induced strong β3-mediated cAMP activation, suggesting more effective downstream signaling and higher potency compared to the other agents evaluated [167]. Moreover, vibegron appears resistant to functional desensitization and may act as an efficacy-dominant agonist capable of sustaining receptor activation over time. In the treatment of chronic conditions such as obesity, this characteristic might prove particularly beneficial. Similarly, Brucker et al. conducted a comparative analysis of vibegron and mirabegron and reported that vibegron demonstrated near-exclusive β3 selectivity along with a higher maximal β3 response. In contrast, mirabegron exhibited partial activity at β2 receptors and minor β1 interaction at supratherapeutic concentrations [168]. These distinctions highlight the potential of vibegron to offer an improved safety and tolerability profile, particularly in long-term clinical use.

A recent network meta-analysis comparing the safety profiles of vibegron and mirabegron in patients with overactive bladder found that both agents were generally well tolerated, but key differences emerged. Vibegron was not associated with increased risk of adverse events compared to placebo, including dry mouth, constipation, hypertension, urinary tract infections, and cardiovascular events. In contrast, mirabegron showed a higher incidence of nasopharyngitis and cardiovascular adverse events relative to placebo. Furthermore, vibegron does not exhibit clinically significant CYP450 enzyme inhibition, particularly CYP2D6, thereby reducing the likelihood of drug–drug interactions. These findings suggest that vibegron may confer a more favorable safety profile for sustained therapeutic use. Its potential applicability in the management of obesity is of particular interest, given the chronic nature of the disease and its frequent association with multiple comorbid conditions that might require polypharmacy. Under such circumstances, cardiovascular safety and metabolic neutrality emerge as significant therapeutic advantages [169].

Additionally, real-world adherence data also favor vibegron. In a large retrospective claims study, patients on vibegron demonstrated modestly but significantly higher adherence and persistence compared to those on mirabegron. Mean proportion of days covered (PDC) was 0.67 for vibegron versus 0.64 for mirabegron; the proportion of adherent patients (PDC ≥ 0.80) was 49.0% versus 45.1%, and the median treatment persistence was 171 versus 128 days, respectively [166]. Although the differences are modest, they may hold clinical relevance for chronic indications such as obesity, where long-term adherence is critical. Nonetheless, as these findings originate from an overactive bladder population, their extrapolation to metabolic disorders warrants careful interpretation.

Both mirabegron and vibegron exemplify the gap between mechanistic rationale and clinical translation in the pharmacologic targeting of thermogenic adipose tissue. While mirabegron has provided critical proof-of-concept for β3-AR–mediated BAT activation in humans, its dose-limiting cardiovascular effects have constrained broader therapeutic application in metabolic disease [49,167,169]. Vibegron, characterized by greater β3 selectivity and a more favorable safety profile, may represent a more promising candidate in this context [167,168]. Nevertheless, its clinical development to date has been restricted to urological indications, and its metabolic effects remain largely unexplored in human studies. This is particularly notable given the current therapeutic landscape, in which approved pharmacologic treatments for obesity primarily target energy intake via central appetite modulation or gastrointestinal mechanisms, with no agents currently approved that directly enhance energy expenditure via adipose tissue activation [156,170]. The absence of such therapies highlights a critical unmet need. Future efforts should focus not only on identifying suitable pharmacologic candidates but also on addressing delivery challenges, particularly those related to tissue specificity and systemic tolerability, to fully realize the therapeutic potential of β3-agonist strategies in obesity management.

## 4. Preclinical vs. Clinical Studies

Early clinical trials of novel selective β3-AR agonists demonstrated limited benefits and issues with consistent bioavailability. A study of the CL 316,243 in young healthy lean men demonstrated increased insulin-mediated glucose disposal due to an increase in nonoxidative glucose disposal and increased fat oxidation; however, there was no change in body weight or composition [171]. Treatment with L-796568 in overweight and obese men showed decreased triacylglycerol levels but did not affect 24 h energy expenditure or glucose tolerance [172]. TAK-677 was studied in 65 obese men and women and showed a small increase in 24 h energy expenditure, but no change in weight, glucose metabolism, or 24 h respiratory quotient [173]. The studies of CL 316,243 and TAK-677 also indicated issues with consistent bioavailability and increased heart rate as an adverse side effect at higher doses [171,173].

With the approval of mirabegron in 2012 for the treatment of overactive bladder, a β3-AR agonist became available that has consistent bioavailability and, at therapeutic doses, causes fewer adverse side effects. Here, we review preclinical and clinical studies conducted on the effects of mirabegron on mechanisms related to BAT activity and thermogenesis.

### 4.1. Preclinical Studies

In mouse brown preadipocytes, mirabegron dose-dependently increased UCP1 expression during differentiation [107]. Similarly, in 3T3-L1 adipocytes, mirabegron upregulated UCP1 together with PGC-1α and Transmembrane Protein 26 (TMEM26), a beige adipocyte marker [107]. Consistent with these findings, Dehvari et al. (2020) reported that mirabegron increased cAMP and UCP1 mRNA in primary brown adipocytes, and likewise induced UCP1 expression in brite adipocytes [174]. In vivo, treatment of high-fat diet–fed mice with mirabegron increased UCP1 mRNA and protein, along with higher expression of Cell Death-Inducing DNA Fragmentation Factor Alpha-Like Effector A (CIDEA), a BAT marker, and Carnitine Palmitoyltransferase 1 (CPT1), the key mitochondrial enzyme that controls fatty acid entry for oxidation [107]. In contrast, Peres Valgas da Silva et al. (2021) found that mirabegron increased UCP1 protein in BAT but not in inguinal WAT, suggesting that its thermogenic effects were mediated predominantly through BAT activation rather than robust inguinal WAT beiging [175]. In a study of obese mice treated with mirabegron via interscapular infusion, the interscapular BAT of mirabegron-treated mice had fewer and smaller lipid droplets compared to control mice. Additionally, there was an increased abundance of multilocular adipocytes in the inguinal WAT [170]. da Silva (2021) [175] similarly noted that the treatment of obese mice with mirabegron prevented obesity-mediated accumulation of lipids in BAT that was found to occur in the control group. However, while there was a partial decrease in inguinal WAT in the mirabegron-treated mice, no increase in multilocular lipid droplets within inguinal WAT was identified [175].

Multiple studies on obese mice have shown that exposure to mirabegron leads to lower body weight [170,175,176]. Hao et al. (2019) [170] found that mirabegron-treated obese mice had lower body weight and lower weight of inguinal WAT, gonadal WAT, and retroperitoneal WAT, although food intake was the same. Another study of obese mice treated with mirabegron orally for two weeks found a decrease in epididymal fat, but no change in body weight [177].

Studies of mice treated with mirabegron have also indicated improvement in glucose metabolism, with improved glucose tolerance and increased insulin sensitivity [170,174,175]. Additionally, mirabegron-treated mice have demonstrated increased energy expenditure and increased interscapular temperature as measured by thermal imaging [175].

Mirabegron treatment of obese mice through multiple drug delivery methods has been found to be effective in influencing metabolic processes. Delivery of mirabegron via the oral route [175], infusion into the interscapular BAT [170], and microneedle patch applied to the inguinal region [176] all resulted in decreased body weight and WAT mass.

Several studies have focused on determining the type of β receptors that act on BAT by knocking out and silencing different β receptors. Dehvari et al. (2020) [174] injected mice with a single intraperitoneal dose of mirabegron and found that mirabegron-treated mice had increased 3H-2-deoxyglucose uptake into BAT and inguinal WAT, improved glucose tolerance, and increased whole body oxygen consumption. These findings were significantly decreased in β3-AR knockout mice [174].

Riis et al. (2020) [178] studied the effects of β-adrenergic stimulation of BAT from human deep neck adipose tissue biopsies and a human BAT cell model. In the cell model, nonselective stimulation of β-AR and selective stimulation of β1-AR led to increased UCP1 expression, whereas selective stimulation of β3-AR by mirabegron and CL 316,243 did not increase UCP1 expression. Silencing of β1-AR significantly decreased these findings. Additionally, in the BAT of the fresh human biopsies, β1-AR mRNA was the highest expressed β receptor, whereas only minor expression of β2-AR and β3-AR was found, with no functional consequences of stimulating β3-AR identified [178].

However, Cero et al. (2021) [56] studied human adipocytes from supraclavicular neck fat and immortalized brown/beige adipocytes from deep neck fat, and expression of β3-AR was detected in preadipocytes and mature adipocytes. Exposure of these tissues to mirabegron stimulated BAT lipolysis and thermogenesis. Silencing of β3-AR significantly decreased this stimulation by mirabegron [56].

### 4.2. Clinical Studies

Clinical studies have been conducted to assess the effect of mirabegron on BAT activity, glucose metabolism, and body composition. Several studies indicate that a single high dose (200 mg) of mirabegron in humans can stimulate BAT metabolic activity, as measured by ^18^F-FDG PET/CT [48,179]. A single dose of 200 mg of mirabegron also increased resting metabolic rate (RMR) among study subjects, with increased BAT metabolic activity found to be a significant predictor of changes in RMR [48].

Additional findings of the effects of a single dose of mirabegron include increased energy expenditure and resting energy expenditure, increased supraclavicular skin temperature, and an increase in insulin levels, nonsterified fatty acids, and lipid oxidation [159,179,180]. Multiple studies indicate that a single dose of mirabegron 200 mg leads to an increase in heart rate and systolic blood pressure, while lower doses have a lesser effect on these parameters [48,159,179,181].

Dose-dependent increases in BAT metabolic activity have been observed with increased BAT activity at the maximal allowable dose (200 mg) as compared to the therapeutic dose (50 mg) [179]. Significant increases in BAT oxidative metabolism, blood flow, and glucose uptake were identified after a single dose of 200 mg of mirabegron, while these changes were not seen at a single dose of 50 mg [181]. The dose-responsiveness of the effects of mirabegron on cardiovascular parameters such as heart rate and blood pressure may be due to non-selective stimulation of β1-AR or β2-AR at high doses of mirabegron, which raises the question of whether the dose-dependent acute increases in BAT metabolic activity found in these studies may be due to non-selective stimulation of β1-AR or β2-AR.

While these studies observe the acute effects of a single dose of mirabegron, several studies examine the metabolic and thermogenic effects of mirabegron in humans over several weeks of treatment. O’Mara et al. (2020) [49] studied fourteen healthy women who took mirabegron 100 mg daily for 4 weeks. Mirabegron-treated subjects demonstrated increased resting energy expenditure and increased BAT metabolic activity and BAT volume as indicated by ^18^F-FDG PET/CT, with the largest increases in BAT activity and volume observed in participants who initially had less BAT at baseline. Glucose metabolism also improved for participants, with findings of increased insulin sensitivity, increased glucose effectiveness, and increased insulin secretion. However, these benefits came at the expense of increases in resting heart rate and systolic blood pressure during treatment [49].

Finlin et al. (2018) [182] tested a lower dose of 50 mg daily but over a longer period of 10 weeks in six obese participants. In this setting, mirabegron increased protein levels of UCP1, TMEM26 and CIDEA in biopsies of abdominal subcutaneous WAT, while no change in PGC-1α was identified. Importantly, unlike O’Mara’s higher-dose, shorter trial, this study did not observe changes in blood pressure or heart rate in the study subjects [182].

Building on these findings, Finlin et al. (2020) [183] extended their work to determine whether chronic mirabegron treatment could induce broader metabolic benefits beyond beige adipocyte formation. This follow-up study targeted an at-risk population, with body mass index >27 and either prediabetes or metabolic syndrome. Treatment with mirabegron at 50 mg daily for 12 weeks did not alter BAT glucose uptake or volume as measured by ^18^F-FDG PET/CT. However, it was associated with improvements in glucose metabolism, including enhanced oral glucose tolerance, insulin sensitivity and β cell function, as well as decreased HbA1c. Consistent with their earlier report, abdominal subcutaneous WAT biopsies again demonstrated increased expression of UCP1, TMEM26, and CIDEA, supporting an effect on adipose tissue beiging. Heart rate and blood pressure were unchanged during the treatment period [183].

Taken together, these studies indicate that mirabegron treatment promotes adipose tissue beiging, as evidenced by increased expression of UCP1, TMEM26, and CIDEA, improves glucose metabolism, and, at higher doses, enhances BAT activity and volume. Notably, however, despite these favorable effects, none of the studies reported a significant change in body weight [49,182,183]. This lack of effect may be attributed to the relatively short treatment durations, modest sample sizes, and limitations in dosing due to cardiovascular safety considerations.

## 5. Conclusions and Future Directions

Obesity remains a global health crisis, and while incretin-based therapies such as GLP-1 receptor agonists have transformed treatment, their primary reliance on appetite suppression and delayed gastric emptying alone often leads to a plateau in weight loss. This limitation highlights the need for complementary approaches that enhance energy expenditure. Thermogenic strategies, including β_3_-adrenergic receptor activation, TRPV1 agonism, mitochondrial uncoupling, and thyroid hormone receptor modulation, represent promising avenues to augment incretin therapies by directly targeting adipose tissue metabolism.

Among these, β_3_-adrenergic receptor agonists have shown particular potential. Agents such as mirabegron can stimulate lipolysis, activate BAT thermogenesis, and improve glucose metabolism, but their translation to obesity treatment has been hindered by low receptor density in adult human BAT and dose-dependent cardiovascular side effects. Vibegron, a next-generation β_3_-agonist with enhanced receptor selectivity and safety, may offer a more favorable profile; however, clinical evidence in metabolic disease remains limited. Importantly, no FDA-approved pharmacotherapies currently exist that directly and safely enhance thermogenesis for the management of obesity.

Future progress will require the development of selective, tissue-specific β_3_-AR agonists, exploration of combination strategies that unite incretin therapy with thermogenic agents, and greater integration of pharmacotherapy with lifestyle interventions and precision medicine approaches. By bridging appetite regulation with energy expenditure, such efforts may achieve more durable, effective, and equitable outcomes in the treatment of obesity.

## Figures and Tables

**Table 1:** Safety concerns are associated with selected thermogenic agents: The table summarizes representative compounds investigated for thermogenic or brown adipose tissue (BAT), including activating effects, their primary mechanisms of action, reported adverse effects, and current development or regulatory status. ↑ indicates an increase in levels, signaling, rate of metabolism, or catecholamine release.

Compound Name	Mechanism of Action	Observed Side Effects	Development Stage (and/or FDA) Status
Mirabegron Vibegron	β3-AR agonist	Increased heart rate [88], hypertension [89], urinary tract infections [90]	FDA-approved for overactive bladder
CL-316,243	β3-AR agonist	Decrease in motor activity [91] decreased cardiac contractility [92]	Preclinical stage, not approved for human use
DNP * (*2,4-Dinitrophenol*)	Mitochondrial uncoupler	Hyperthermia [93], tachycardia [94], fatal toxicity [60]	Not approved by the FDA, banned due to safety concerns
/TRPV1 agonists *	TRPV1 receptor- activation stimulates sympathetic system	Inflammation of GI tract [95], hyper- [96] or hypotension [97] myocardial infarction [98]	Qutenza (8% capsaicin) is FDA-approved for neuropathic pain
Thyroid hormone analogs (e.g., GC-1) *	TH receptor activation leads to ↑ metabolism & UCPs	Tachycardia [99], arrhythmias [99], muscle and bone mass loss [100]	Sobetirome (GC-1) drug development halted due to cardiac risks
Centrally acting sympathomimetics *	↑ hypothalamic catecholamine release	Dry mouth [101], constipation, insomnia [102]	Phentermine is FDA-approved for short-term weight management

* A thermogenic agent that is an alternative to β3-adrenergic receptors, mechanisms, or pathways.

## Data Availability

No new data were created or analyzed in this study. Data sharing is not applicable to this article.

## Abbreviations

The following abbreviations are used in this manuscript:

AC	Adenylyl cyclase
AMPK	5′adenosine monophosphate-activated protein kinase
AR	Adrenergic receptors
ATGL	Adipose triglyceride lipase
ATP	Adenosine triphosphate
BAT	Brown adipose tissue
cAMP	Cyclic adenosine monophosphate
CIDEA	Cell death–inducing DFFA-like effector A
CREB	cAMP-responsive element-binding protein
DNP	2,4-dinitrophenol
EE	Energy expenditure
FDA	U.S. Food and Drug Administration
FFA	Free fatty acid
GCPR	G protein-coupled receptors
GIP	Glucose-dependent insulinotropic polypeptide
GIPR	GIP receptor
GLP–1 RA	GLP-1 receptor agonist
GLP-1	Glucagon-like peptide-1
GLP-1R	GLP-1 receptor
Gs	Stimulatory G protein
HSL	Hormone-sensitive lipase
MGL	Monoacylglycerol lipase
PGC	1α-Peroxisome proliferator-activated receptor gamma coactivator 1-alpha
PKA	Protein kinase A
RMR	Resting metabolic rate
SNS	Sympathetic nervous system
TAG	Triacylglycerol
TH	Thyroid hormone
THR	Thyroid hormone receptor
TMEM26	Transmembrane protein 26
UCP1	Uncoupling protein 1
WAT	White adipose tissue
WHO	World Health Organization
β3-AR	Beta-3 adrenergic receptors
^18^F-FDG	^18^F-fluorodeoxyglucose
^18^F-FDG PET/CT	^18^F-fluorodeoxyglucose positron emission tomography/computerized tomography
